# Laboratory and Semi-Field Cage Demography Studies of *Diachasmimorpha longicaudata* Mass-Reared on Two *Ceratitis capitata* Strains

**DOI:** 10.3390/insects16101031

**Published:** 2025-10-06

**Authors:** Lorena Suárez, Segundo Ricardo Núñez-Campero, Silvia Lorena Carta Gadea, Fernando Murúa, Flávio Roberto Mello Garcia, Sergio Marcelo Ovruski

**Affiliations:** 1 Plant, Animal, and Food Health Bureau of the Government of the San Juan Province, Nazario Benavides 8000 Oeste, Rivadavia, San Juan J5413ZAD, Argentina; silorecarta@gmail.com (S.L.C.G.); fmurua80@gmail.com (F.M.); 2 CCT CONICET San Juan, Avenida Libertador General San Martín 1109, San Juan J5400AR, Argentina; 3 La Rioja Regional Center for Scientific Research and Technology Transfer (CRILAR-CONICET), Entre Ríos y Mendoza s/n, Anillaco, La Rioja F5301, Argentina; segundo.nc@conicet.gov.ar; 4 Department of Exact, Physical and Natural Sciences, Institute of Conservation Biology and Paleobiology (IBICOPA), National University of La Rioja (UNLaR), Avenida Luis de la Fuente s/n, La Rioja F5300, Argentina; 5 Department of Ecology, Zoology and Genetics, Institute of Biology, Federal University of Pelotas, Pelotas 96000, RN, Brazil; flavio.garcia@ufpel.edu.br; 6 Microbiological Industrial Processes and Biotechnology Pilot Plant (PROIMI-CONICET), Biological Control Department, Avenida Belgrano y Pasaje Caseros, San Miguel de Tucumán T4001MVB, Argentina; sovruski@conicet.gov.ar

**Keywords:** Mediterranean fruit fly, parasitoid mass rearing, demographic parameters, rearing quality control parameters, fruit fly biological control

## Abstract

**Simple Summary:**

The reproductive capacity of parasitoid wasps during their lifetime plays a crucial role in understanding their potential as biocontrol agents and the host–parasitoid dynamics. An interesting system to study involves the Southeast Asia-native parasitoid *Diachasmimorpha longicaudata* and its host *Ceratitis capitata*, commonly known as the Mediterranean fruit fly or medfly, which is a serious invasive fruit fly pest in Argentina. This study compared reproductive parameters of two parasitoid population lines reared at the biofactory San Juan on different medfly strains. One line was mass-reared on medfly larvae of a genetic sexing strain (=*Dl*_(*Cc*-tsl)_) and the other on larvae of a wild biparental medfly strain (=*Dl*_(*Cc*-bip)_). The goal was to provide information for improving parasitoid mass production and evaluating its performance under natural conditions. For this, laboratory and semi-field cage trials were conducted at the San Juan Biofactory. Firstly, laboratory trials showed that *Dl*_(*Cc*-bip)_ females displayed higher reproductive and population rates than those of *Dl*_(*Cc*-tsl)_ females. Secondly, semi-field cage trials revealed that females of both *Dl*_(*Cc*-bip)_ and *Dl*_(*Cc*-tsl)_ had similar and high reproductive potential in late spring and summer, when environmental conditions are temperate–warm. However, unlike *Dl*_(*Cc*-tsl)_ females, *Dl*_(*Cc*-bip)_ females were reproductively active in early autumn, albeit at very low rates due to colder environmental conditions. The current study provides novel data to improve the productivity of *D. longicaudata* mass rearing and to achieve the most effective medfly control through parasitoid releases in the semi-arid, fruit-growing areas of Argentina.

**Abstract:**

*Ceratitis capitata* (Wiedemann) or medfly is a polyphagous pest of fruit crops worldwide. The Asian-native larval parasitoid *Diachasmimorpha longicaudata* (Ashmead) is mass-reared at the San Juan Biofactory and is currently released for medfly control in Argentina. Information on parasitoid survival, reproduction, and population growth parameters is critical for optimizing the mass-rearing process and successfully achieving large-scale release. This study provides a first-time insight into the demography of two population lines of *D. longicaudata*: one mass-reared on medfly larvae of the Vienna-8 temperature-sensitive lethal genetic sexing strain and the other on larvae of the wild biparental medfly strain. The aim was to compare both parasitoid populations to improve mass-rearing quality and to assess performance on medfly in a semi-arid environment, typical of Argentina’s central-western fruit-growing region. Tests were performed under laboratory and non-controlled environmental conditions in semi-field cages during three seasons. *Dl*_(*Cc*-bip)_ females exhibited higher reproductive potential than did *Dl*_(*Cc*-tsl)_ females under lab conditions. However, both *Dl*_(*Cc*-bip)_ and *Dl*_(*Cc*-tsl)_ were found to be similar high-quality females with high population growth rates in warm–temperate seasons, i.e., late spring and summer. *Dl*_(*Cc*-bip)_ females were only able to sustain low reproductive rates in early autumn, a colder season. These results are useful for improving the parasitoid mass production at the San Juan Biofactory and redesigning parasitoid release schedules in Argentina’s irrigated, semi-arid, fruit-growing regions.

## 1. Introduction

In several Latin American countries where fruit production, marketing, and export are affected by tephritid fruit fly species of economic and quarantine importance, such as *Ceratitis capitata* (Wiedemann), *Anastrepha fraterculus* (Wiedemann), *A. suspensa* (Loew), *A. obliqua* (Macquart), *A. striata* Schiner, and *A. ludens* (Loew) (Diptera: Tephritidae), among other fruit fly species, a renewed use of biological control against those pests is currently underway [1,2,3,4]. Among the different natural enemies evaluated are entomopathogenic fungi, nematodes, bacteria, viruses [5], predators, and parasitoid wasps [3], but the last of these comprise the most widely used method as a valid alternative in fruit fly biological control programs [3,6,7,8,9]. This is related to two major linked trends: (1) the development and improvement of successful mass-rearing techniques of exotic and native parasitoids for augmentative releases [10,11,12,13,14,15] and (2) the use of different eco-friendly tactics that preserve biodiversity and reduce agrochemical use [16,17].

The use of a parasitoid species to implement an augmentative biological control program, which involves the large-scale release of mass-reared parasitoids [18], requires the release of high-quality individuals, i.e., adults with higher reproductive potential, longer lifespans, and higher host-searching and parasitism abilities, among other key attributes [19,20,21,22]. Therefore, understanding the demography of fruit fly parasitoids provides useful information about their reproductive and population biology, an essential tool for assessing the performance of different species as biocontrol agents [23,24]. In this regard, demographic parameters can be used to compare the effect of different hosts on the production of parasitoids and thus optimize their rearing on a particular host under artificial conditions [15,25,26,27]. Population growth parameters are also helpful in testing the performance of parasitoid species in controlling target pest populations under different environmental conditions and/or on diverse hosts [28,29,30,31,32,33,34]. Furthermore, an in-depth study on the parasitoid demography enables an interdisciplinary approach involving genetic, evolutionary, and ecological aspects, and environmental factors [35]. In this regard, demographic parameters should be evaluated in relation to the weather variables of the region where the parasitoid species will be released, in order to ensure the viability of the individuals in the new area [36]. Air temperature is an environmental variable predictor of insect development dynamics [37] and is therefore a determining climatic factor for parasitoid establishment and performance in the environment where it is released [38]. Air temperature directly affects the survival and fecundity of parasitoids and also influences the parasitism rate [39,40,41,42,43,44,45].

Such knowledge about parasitoids may help with the successful biological control of the globally invasive *C. capitata* or medfly in Argentina. The medfly is one of the main fruit pests severely constraining exports for Argentinian fruit and vegetable producers, which has a negative socioeconomic cost on the country [46]. This exotic fruit fly pest has spread throughout almost all fruit-growing regions of Argentina. The medfly currently covers a large area of the country from 22° S to 36° S as a result of both its biological plasticity for adapting to different climatic conditions and its broad host range, involving commercial and wild fruit species [47,48]. In this context, medfly control actions have been based on the integrated use of the Sterile Insect Technique (SIT); chemical, cultural, and trapping methods; and quarantine protection systems during the last 30 years [49]. A biological control method was added in 2008 to the integrated management strategies of the San Juan Fruit Fly Control and Eradication Program (ProCEM-San Juan, Spanish acronym) [50]. Such a control tactic was implemented to achieve mass rearing of the Indo-Pacific parasitoid *Diachasmimorpha longicaudata* (Ashmead) (Hymenoptera: Braconidae) at the San Juan Biofactory to release it in fruit-producing semi-arid valleys of the San Juan province, central-western Argentina. The lack of resident parasitoid species attacking medflies in the region [50] supported the introduction and release of *D. longicaudata*, which was released under different environmental conditions [51,52,53,54]. This exotic braconid wasp was originally introduced and released in several Latin American countries, including Argentina, as a classical biological control agent for fruit fly pests between the 1960s and 1980s [3,4,6]. Specimens of *D. longicaudata* were recovered in citrus-growing areas of northern Argentina approximately 40 years after its first release in that region [3]. *Diachasmimorpha longicaudata* is currently one of the most widely used parasitoid species for augmentative biological control of fruit fly pests [3,6,11]. It is a generalist, larval, koinobiont, and solitary endoparasitoid of several tephritid fruit fly species [3,55]. Females of *D. longicaudata* forage on fallen infested fruits and also on fruit still in the tree canopy, and they always oviposit into a host larva by drilling with their ovipositor into the fruit pericarp from outside [56]. There were no records of this exotic parasitoid attacking non-target hosts or beneficial insect species in those American countries where it was released [3].

The mass rearing of *D. longicaudata* was successfully established on irradiated larvae of the medfly Vienna-8 temperature-sensitive lethal genetic sexing strain without inversion (=*Cc*_tsl_ strain) at the San Juan Biofactory between 2011 and 2012, although this strain has high production costs [48]. This medfly strain is currently used for producing sterile males of the pest to apply the SIT in irrigated fruit crops throughout San Juan province [57]. From 2012 to 2016, augmentative releases of *D. longicaudata* were carried out in fruit crops of different fruit-growing areas of the San Juan province to evaluate its performance as a medfly biocontrol agent under semi-arid climatic conditions [51,58]. The use of augmentative biological control in San Juan achieved a medfly population control between 40 and 70% [58]. These promising outcomes encouraged research to improve both the mass production of *D. longicaudata* and the quality of its individuals yielded at the San Juan Biofactory.

Given this, it was first hypothesized that the use of larvae of a biparental medfly strain native to San Juan (=*Cc*_bip_ strain) as a host to rear *D. longicaudata* instead of the *Cc*_tsl_ strain enhances and optimizes the parasitoid production, providing individuals with higher reproductive capacity. Therefore, the first aim of this study was to compare relevant population and reproductive parameters under laboratory rearing conditions between two *D. longicaudata* population lines, one reared on the *Cc*_bip_ strain and the other on the *Cc*_tsl_ strain. Secondly, it was hypothesized that population lines of *D. longicaudata* reared on either of the two medfly strains perform better, based on their reproductive success, when released during the spring–summer period. Thus, the second aim of this study was to compare the parasitoids’ performance of the two *D. longicaudata* population lines among three seasons, namely spring, summer, and autumn, under natural weather conditions in the area of interest using semi-field cages. During such seasons, the medfly is particularly active in irrigated fruit-growing valleys of San Juan due to the abundance of different host fruit species [59]. Among host plants, peaches and figs, which occur between late spring and mid-summer, are multiplying fruits for *C. capitata* populations in the study region [50,58,59]. In addition, large fruits, such as citrus fruits, particularly oranges and grapefruits, can support *C. capitata* populations during less favorable environmental periods, such as autumn and winter [48,58]. In this regard, it is a significant challenge for the exotic *D. longicaudata* to adapt to the fruit-growing region due to its temperate and semi-arid climatic characteristics, and its broad thermal variations between seasons, and also throughout the day. The significance of this study is discussed regarding the use of *D. longicaudata* in augmentative biological control against fruit fly pests in Argentina.

## 2. Materials and Methods

### 2.1. Insect-Rearing Procedures

Parasitoids and flies came from colonies kept in the San Juan Insect Mass Rearing Biofactory, which belongs to the Plant, Animal, and Food Health Bureau (= PAFHB) of the government of the San Juan province, located in the central-western fruit-growing region of Argentina. Two *D. longicaudata* population lines (from now on, *Dl*PLs) were used in the trials. One of them was reared on *C. capitata* third-instar larvae of the *Cc*_tsl_ strain (from now on, *Dl*_(*Cc*-tsl)_) and the other one on *C. capitata* third-instar larvae of the *Cc*_bip_ strain (from now on, *Dl*_(*Cc*-bip)_). The *Cc*_tsl_ strain was established at the San Juan Biofactory in the early 2000s, which is currently reared. This medfly strain was brought from the Km-8 Pilot Biofactory located in the neighboring province of Mendoza, but originally the strain was sent by the International Atomic Energy Agency. Such a strain is currently reared through different colonies at the Medfly Rearing Laboratory (=MRL) from the San Juan Biofactory. Egg incubation begins at moderate temperatures, i.e., 24 °C, which allows for hatching and larval development on a suitable diet (described below). The first colony is the “Filter” colony, where the most suitable adults are manually selected for reproduction. The eggs from this colony give rise to the second colony of females, the “Injection” colony, whose eggs originate the third colony, the “Renewal” colony, which in turn produces the fourth colony, the “Release” colony. This then gives rise to the last colony, the “Thermal” colony, where the eggs undergo heat treatment at restrictive high temperatures, i.e., between 34 and 35 °C, for 48 h to selectively eliminate heterozygous females. As a result, a male-only population emerges from brown pupae, which are irradiated for use in SIT programs. The *Cc*_bip_ strain originated from wild medfly larvae recovered from figs, peaches, and plums collected from orchards in the fruit-growing valley of Tulum, San Juan, between December 2018 and January–February 2019. Larvae of both medfly strains were reared at the MRL on an artificial diet based on wheat bran (17%), yeast (8%), sugar (10%), hydrochloric acid (0.8%), poplar wood chips (8.8%), water (54.9%), and food preservative, such as sodium benzoate (0.3%) and methylparaben (0.2%). The medfly strains were reared in separate rooms, as the *Cc*_tsl_ strain involves a higher degree of complexity for its production. The colonies of the two *Dl*PLs were kept in rectangular iron-framed, voile-covered cages (60 × 60 × 30 cm) at 24 ± 1 °C, 65 ± 5% RH, and at 12:12 (L:D) h, but in different rooms from the Parasitoid Rearing Laboratory at the San Juan Biofactory. Adult parasitoids were provided with pure bee honey and, individually, water through troughs with a yellow absorbent cloth wick every other day. Medfly larvae aged 6 d old and irradiated at 90 Gy were daily exposed to parasitoid females. The irradiation of larvae from both medfly strains was performed using an IMO-1 mobile irradiator with a Co-60 source of γ irradiation, which belongs to the National Atomic Energy Commission from Argentina but is located at the San Juan Biofactory. The larval quality of both medfly strains was evaluated using the average weight of 300 6 d old larvae samples per batch. Batches with 12.2 ± 0.5 mg (Mean ± SE) mean weight larvae were used in the trials as suggested by Suárez et al. [60]. Cohorts from *Dl*_(*Cc*-tsl)_ and *Dl*_(*Cc*-bip)_) colonies at their 60th and 10th generations under artificial rearing conditions were used in the assays.

### 2.2. Experimental Setup

#### 2.2.1. Laboratory Trials

Survival and lifetime reproductive parameters of both *Dl*_(*Cc*-tsl)_ and *Dl*_(*Cc*-bip)_ were assessed and compared under the same lab-controlled conditions described above. Twenty-five female/male pairs of *Dl*_(*Cc*-tsl)_ and *Dl*_(*Cc*-bip)_ were individually placed into transparent cubical Plexiglas cages (10 cm). All 25 pairs of each parasitoid population line remained isolated during their lifetime. Parasitoids were provided with water and honey every other day. Ninety lab-reared 6 d old larvae of the *C. capitata* strain belonging to the respective parasitoid population line, i.e., *Cc*_tsl_ and *Cc*_bip_, were placed inside artificial units and exposed to each parasitoid pair for 2 h under a lighting condition of 1200 lux provided by six 36W-fluorescent light tubes distributed throughout the room. The oviposition devices were 5 × 0.7 cm (diameter × height) voile screen-covered plastic dishes holding naked irradiated host larvae, i.e., no larval-rearing diet. Larval exposure was performed every other day until all female parasitoids died. After exposure to parasitoids, host larvae were removed from each oviposition device and placed in 8 × 7 cm (height × diameter) voile-covered plastic cups with poplar shaving (*Populus alba* L., Salicaceae) at the bottom as a pupation substrate. Puparia were kept inside cups until adult parasitoid emergence. Standard life tables were developed in order to calculate demographic parameters such as *lx*, the proportion of individuals surviving to start of the age interval; *px*, the proportion of individuals surviving through the period; *qx*, proportion of individuals dying through the period; *dx*, the fraction of the original cohort dying at age x; *ex*, life expectancy of individuals surviving at age x; and *mx*, female offspring produced per female at age x [61]. In this study, *lx* and *ex* were calculated based on surviving females (= *lx*_f_ and *ex*_f_, respectively). Based on basic life table parameters, key population increase parameters were calculated such as *R*_0_, net reproductive rate or contribution of newborn females by progeny to the next generation; *r*, intrinsic rate of natural increase or rate of natural increase in a closed population; *λ*, finite rate of increase or rate at which a population increases from time t to time t + 1; and *T*, mean generation time or average time needed for a newborn female to replace herself *R*_0_-times [61].

#### 2.2.2. Semi-Field Cage Trials

Survival and lifetime reproductive rates of both *Dl*_(*Cc*-tsl)_ and *Dl*_(*Cc*-bip)_ were assessed and compared inside a 3.5 × 3.0 m (diameter × height) nylon semi-field cage under natural weather conditions at an experimental plot of the PAFHB, located at 31°31′ S, 68°36′ W, and 710 m.a.s.l. in the Tulum Valley, San Juan province. Such San Juan lowlands have a semi-desert climate with a mean annual temperature of ~18 °C, and rainfall is restricted to the early and mid-summer (January–February) [62]. Mean air temperature was calculated by averaging daily maximum and minimum temperatures, mean maximum air temperature, mean minimum air temperature, and mean relative humidity for each season in the months during which the tests were performed (Table 1). A digital weather station (LUFT^®^, model WS80, Shenzen, China) located in the experimental plot was used for this purpose.

The semi-field cage was placed under poplar trees, providing a windbreak and a constant natural shade. In spring, the study was carried out from October to November 2019, in summer between February and March 2020, and in autumn during May 2020. This design provided a comparison of population and reproductive parameters of both *Dl*PLs under meteorological variations in the same season and between seasons. Forty 15 × 20 cm (diameter ×height) experimental cylindrical iron-framed, voile-covered cages (= ECs) were placed into the semi-field cage. A total of 20 ECs held one female–male pair of the *Dl*_(*Cc*-tsl)_ population line each, whereas the other 20 ECs held one female–male pair of the *Dl*_(*Cc*-bip)_ each. All ECs were placed on a table in the center of the field cage and 1 m above the ground. Every 24 h, the position of each EC was changed in a clockwise rotation. Parasitoids were provided with water and bee honey daily. The oviposition units are described above. Ninety lab-reared 6 d old larvae of *Cc*_tsl_ or *Cc*_bip_ were exposed to *Dl*_(*Cc*-tsl)_ or *Dl*_(*Cc*-bip)_ females, respectively, in previously described oviposition units for 2 h. Larval exposure was held from 10 to 12 AM every other day for 19 days only. In each EC, the survival of individuals was recorded daily. Dead parasitoids were removed to avoid fungal and/or bacterial contamination. Once the exposure period was over, host larvae were handled and kept as previously reported. Standard life tables were developed in order to calculate key population parameters as previously explained.

### 2.3. Data Analysis

Life tables, population parameters, survival, fecundity, and relationships between weather predictors and both *lx*_f_ and *mx* parameters were analyzed using the R-4.4.2 software [63]. Life tables were constructed for both *Dl*_(*Cc*-bip)_ and *Dl*_(*Cc*-tsl)_ using experimental data on mean fecundity per maternal female age interval and female survivorship proportion with the *tidyverse* [64] and *boot* [65,66] packages. Data preprocessing included transformations via mutate() and grouping with group_by() to calculate relative age (*x*) from dates, *mx*, and *lx*_f_. Key population parameters, such as *R*_0_, *r*, *λ*, and *T*, were computed via custom-defined functions. Standard errors (SE) and 95% confidence intervals (CIs) for each population parameter were estimated through stratified bootstrap resampling (R = 1000), significant differences in the population parameters of both strains were established through confidence interval analysis, thereby avoiding inferences derived from bootstrap-generated datasets according to Pritchard et al. [67]. The *ex*_f_ was derived from the cumulative sum of *lx*_f_ (*Tx*), and *lx*_f_, *ex*_f_, and *mx* curves were plotted using *ggplot2* [68]. Log-Rank tests were performed using the *survival* package [69,70] to compare *lx* curves for *Dl*_(*Cc*-bip)_ and *Dl*_(*Cc*-tsl)_. Additionally, a linear model with interaction was fitted, and the effect of both *Cc*_tsl_ and *Cc*_bip_ strains on curve shape was assessed via ANOVA. Both *mx* and *ex* parameters for *Dl*_(Cc-bip)_ and *Dl*_(Cc-tsl)_ were compared using a Generalized Additive Model (GAM) fitted with the *mgcv* package [71]. The model included both *Cc*_tsl_ and *Cc*_bip_ strains as a categorical predictor and female age as a smoothed continuous term, estimated via restricted maximum likelihood (REML). This approach enabled flexible modeling of age-dependent fecundity and life expectancy patterns while accounting for differences due to parasitoids reared on *Cc*_tsl_ or *Cc*_bip_ strains. Model diagnostics and visualization of the smooth term were performed using built-in functions from *mgcv*. The influence of weather predictors, derived from temperature and relative humidity, was evaluated on *lx* and *mx* of both *Dl*_(Cc-bip)_ and *Dl*_(Cc-tsl)_. Data were analyzed employing the *ggplot2*, *dplyr* [72], *tidyr* [73], *relaimpo* [74], *betareg* [75], *car*, and *mgcv* [76] packages. Initial models included all weather variables (T_max_, T_min_, T_mean_, T_range_, RH_max_, RH_min_, RH_mean_, and RH_range_) and were individually fitted for *Dl*_(Cc-bip)_ and *Dl*_(Cc-tsl)_ using linear regression. Model selection was performed via stepwise procedures based on Akaike’s Information Criterion (AIC) to identify the most informative predictors. The relative importance of variables was assessed using the LMG method. A beta-regression approach was applied for *lx*_f_, due to the continuous and bounded nature (0–1) of the variable [77]. Collinearity diagnostics were conducted using variance inflation factors, and non-identifiable terms were excluded. Final models incorporated female age and selected weather variables, which provide a sound basis for making inferences about survival effects.

## 3. Results

### 3.1. Life Table and Population Increase Parameters Under Laboratory Conditions

**Figure 1:**
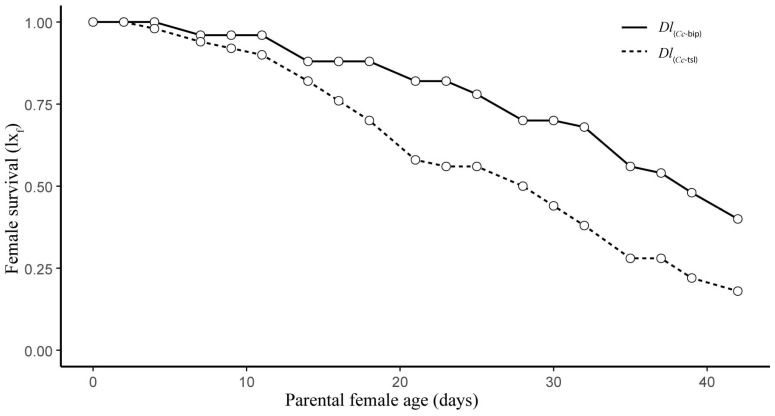
Female survival (*lx*_f_, number of females to start the age interval x/initial number of females) for two population lines of *D. longicaudata* (*Dl*_(*Cc*-tsl)_ and *Dl*_(*Cc*-bip)_).

The life table parameters recorded for both *Dl*PLs are presented in File S1. The survival of females from both *Dl*PLs was not significantly different (Log-rank *χ*^2^ = 3.4, *df* = 1, *p* = 0.07) (Figure 1). The 50% of *Dl*_(*Cc*-bip)_ and *Dl*_(*Cc*-bip)_ females were alive (*l*_50_) at 39 and 29 days, respectively (Figure 1).

**Figure 2:**
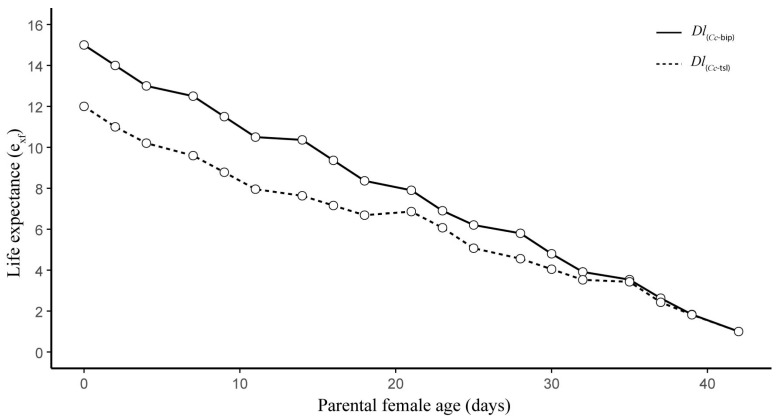
Female life expectancy (*ex*_f_) life expectancy of females surviving at age x for two population lines of *D. longicaudata* (*Dl*_(*Cc*-tsl)_ and *Dl*_(*Cc*-bip)_).

The life expectancy of *Dl*_(*Cc*-bip)_ females was significantly higher than that of *Dl*_(*Cc*-tsl)_ females (*F*_(3, 34)_ = 1701, *p* < 2.2 × 10^−16^, adjusted *R*^2^ = 0.993) (Figure 2). This variation between both *Dl*PLs was influenced by the statistical significance of the parasitoid female’s age (*F* = 4777.64, *df* = 1, *p* = 0.000), the *Dl*PL (*F* = 217.93, *df* = 1, *p* = 0.000), and the interaction between both fixed factors (*F* = 106.32, *df* = 1, *p* = 0.000). Female life expectancy was 15 and 12 days for *Dl*_(*Cc*-bip)_ and *Dl*_(*Cc*-tsl)_, respectively, at the time of adult emergence.

**Figure 3:**
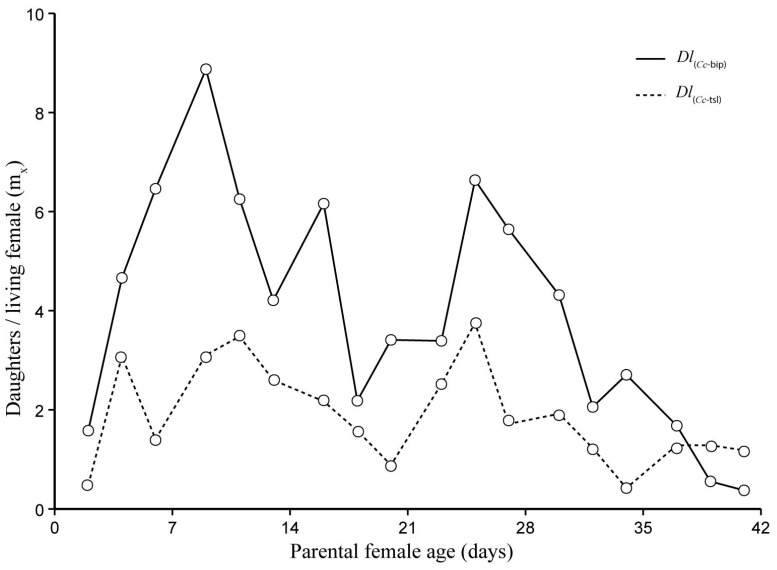
Daily fecundity (mx, number of daughters/parental female/day) for two population lines of *D. longicaudata* (*Dl*_(*Cc*-tsl)_ and *Dl*_(*Cc*-bip)_).

Both *Dl*PLs showed fecundity curves (*mx*) with relatively similar trends, albeit with significantly different mean values of female offspring per mother per day (Figure 3). Over her lifetime, *Dl*_(*Cc*-tsl)_ produced 1.88 (±0.42) fewer daughters than *Dl*_(*Cc*-bip)_ (*t* = 4.34, *df* = 1, *p* = 0.0001). The age of the parental female was a significant factor influencing the difference between female offspring productions in both *Dl*PLs (*F* = 5.76, e.*df* = 5.84, Ref.*df* = 6.99, *p* = 0.0001, R^2^_(adj)_ = 0.61). The *mx* curves showed three peaks of daughter production, which were at 9, 16, and 25 d old for *Dl*_(*Cc*-bip)_ parental females and at 4, 11, and 25 d old for *Dl*_(*Cc*-tsl)_ parental females (Figure 3). A slightly female-biased offspring sex ratio, 1.3:1 and 1.1:1 females/male, was recorded for *Dl*_(*Cc*-bip)_ and *Dl*_(*Cc*-tsl)_, respectively.

Based on confidence intervals, the population increase parameters *R*_0_, *r*, and *λ* recorded for *Dl*_(*Cc*-bip)_ were higher than those for *Dl*_(*Cc*-tsl)_, whereas *T* was similar in both *Dl*PLs (Table 2).

### 3.2. Life Table and Population Increase Parameters Under Semi-Field Cage Conditions

**Figure 4:**
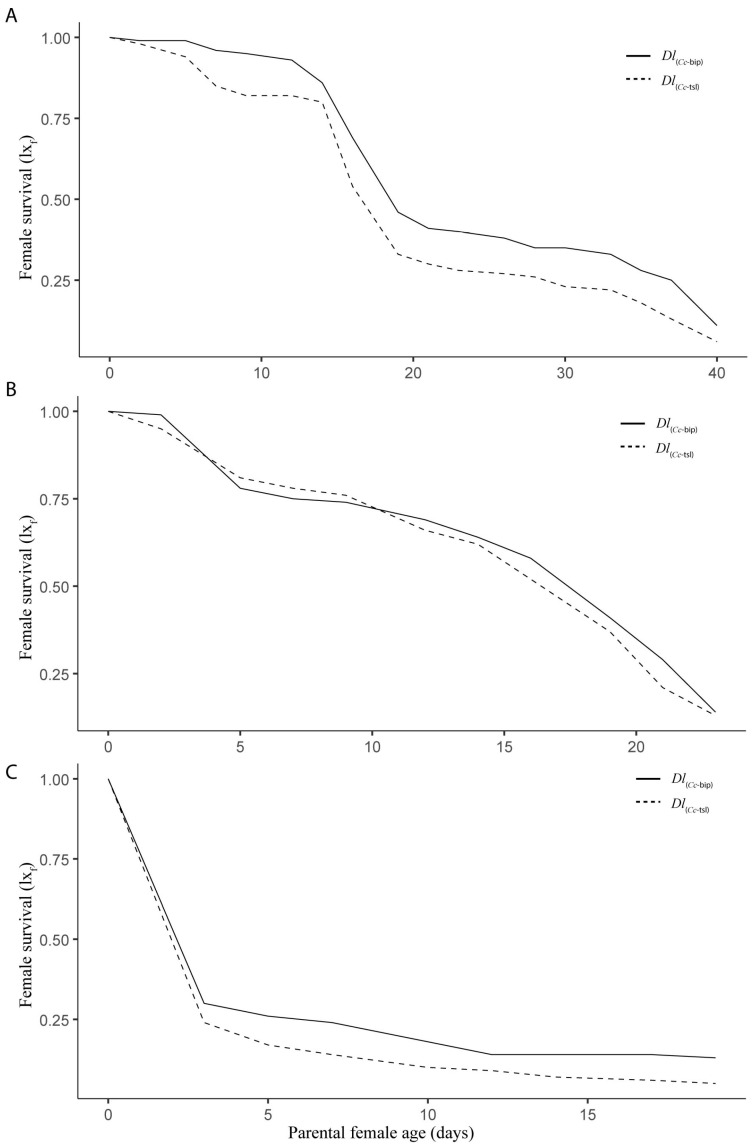
Female survival (*lx*_f_), number of female to start the age interval x/initial number of females) for two population lines of *D. longicaudata* (*Dl*_(*Cc*-tsl)_ and *Dl*_(*Cc*-bip)_) recorded from semi-field cage trials in spring (**A**), summer (**B**), and autumn (**C**) in the Tulum Valley, San Juan Province, central-western Argentina.

The life table parameters recorded for both *Dl*PLs during semi-field cage trials in spring, summer, and autumn are presented in Files S2–S4. Survival curves of females from both *Dl*PLs were significantly different in spring (Log-rank *χ*^2^ = 4.17, *df* = 1, *p* = 0.041) (Figure 4A), but significantly similar in both summer (Log-rank *χ*^2^ = 1.27, *df* = 1, *p* = 0.259) (Figure 4B) and autumn (Log-rank *χ*^2^ = 0.01, *df* = 1, *p* = 0.929) (Figure 4C). The survival rate for *Dl*_(*Cc*-bip)_ was slightly higher than that for *Dl*_(*Cc*-tsl)_ in spring (Figure 4A). Fifty percent of *Dl*_(*Cc*-bip)_ and *Dl*_(*Cc*-bip)_ females were alive (*l*_50_) at 18, 17, and 2 d old, and at 17, 16, and 2 d old in spring, summer, and autumn, respectively. According to the beta regression model, both T_min_ and RH_min_ were weather factors that accurately fitted the model when survival of both *Dl*_(*Cc*-bip)_ and *Dl*_(*Cc*-tsl)_ was analyzed (*Dl*_(*Cc*-bip)_, Log-likelihood = 19.53, *df* = 5, Pseudo-*R*^2^ = 0.6083; *Dl*_(*Cc*-tsl)_ Log-likelihood = 20.96, *df* = 5, Pseudo-*R*^2^ = 0.6584). However, the T_min_ significantly influenced both *Dl*LPs (*Dl*_(*Cc*-bip)_, *z* = 3.029, *p* = 0.00245; *Dl*_(*Cc*-tsl)_, *z* = 3.505, *p* = 0.00046), whereas the RH_min_ did not significantly affect both *Dl*LPs (*Dl*_(*Cc*-bip)_, *z* = 1.779, *p* = 0.07527; *Dl*_(*Cc*-tsl)_, *z* = 1.884, *p* = 0.05962).

**Figure 5:**
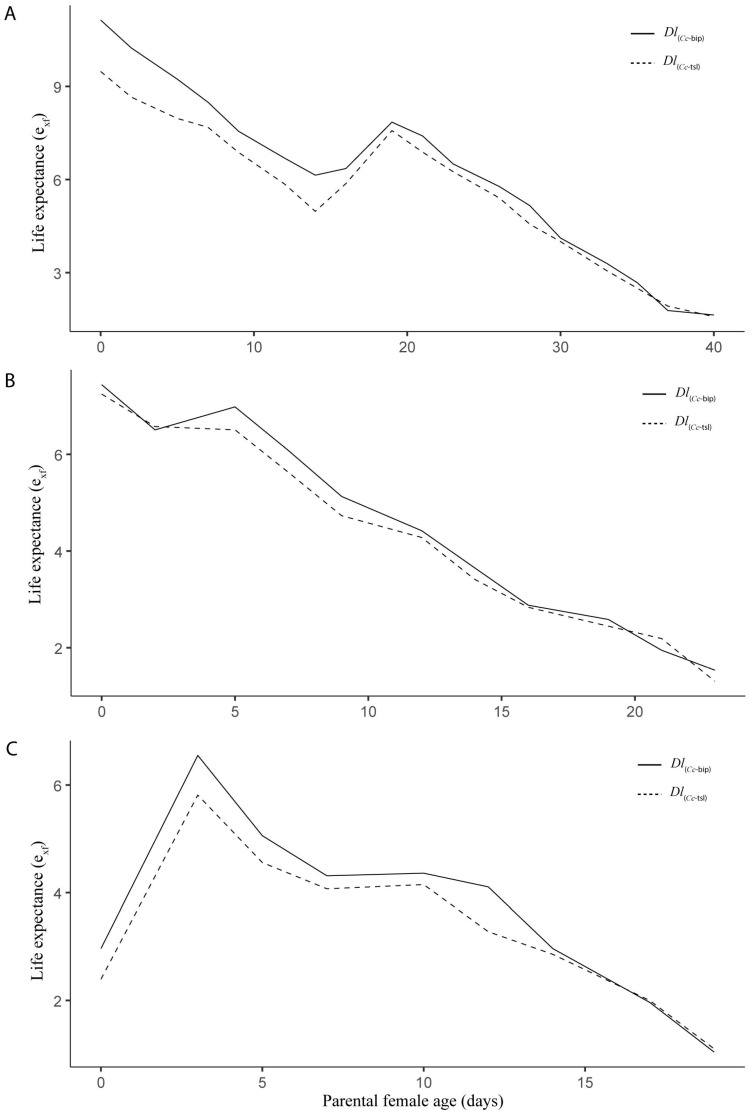
Female life expectancy (*ex*_f_) (life expectancy of females surviving at age x) for two population lines of *D. longicaudata* (*Dl*_(*Cc*-tsl)_ and *Dl*_(*Cc*-bip)_) recorded from semi-field cage trials in spring (**A**), summer (**B**), and autumn (**C**) in the Tulum Valley, San Juan Province, central-western Argentina.

The life expectancy of females between both LPPs was significantly different during semi-field cage trials at three tested stations (spring, *t* = 5.004, *df* = 1, *p* < 0.0001; summer, *t* = 2.892, *df* = 1, *p* = 0.0139; autumn, *t* = 3.156, *df* = 1, *p* = 0.0125). Over *Dl*_(*Cc*-tsl)_ females’ lifespan, the *ex*_f_ was 0.61 (±0.12), 0.18 (±0.06), and 0.34 (±0.11) days significantly lower than that of *Dl*_(*Cc*-bip)_ females during spring (Figure 5A), summer (Figure 5B), and autumn (Figure 5C). The parental female age significantly influenced the life expectancy of both *Dl*PLs females (spring, *F* = 184.9, e.*df* = 8.186, Ref.*df* = 8.817, *p* = 2.0 × 10^−16^, adjusted-*R*^2^ = 0.979; summer, *F* = 436.5, e.*df* = 8.395, Ref.*df* = 8.90, *p* = 2.0 × 10^−16^, adjusted-*R*^2^ = 0.995, autumn, *F* = 89.87, e.*df* = 7.311, Ref.*df* = 7.93, *p* = 2.0 × 10^−16^, adjusted-*R*^2^ = 0.977).

**Figure 6:**
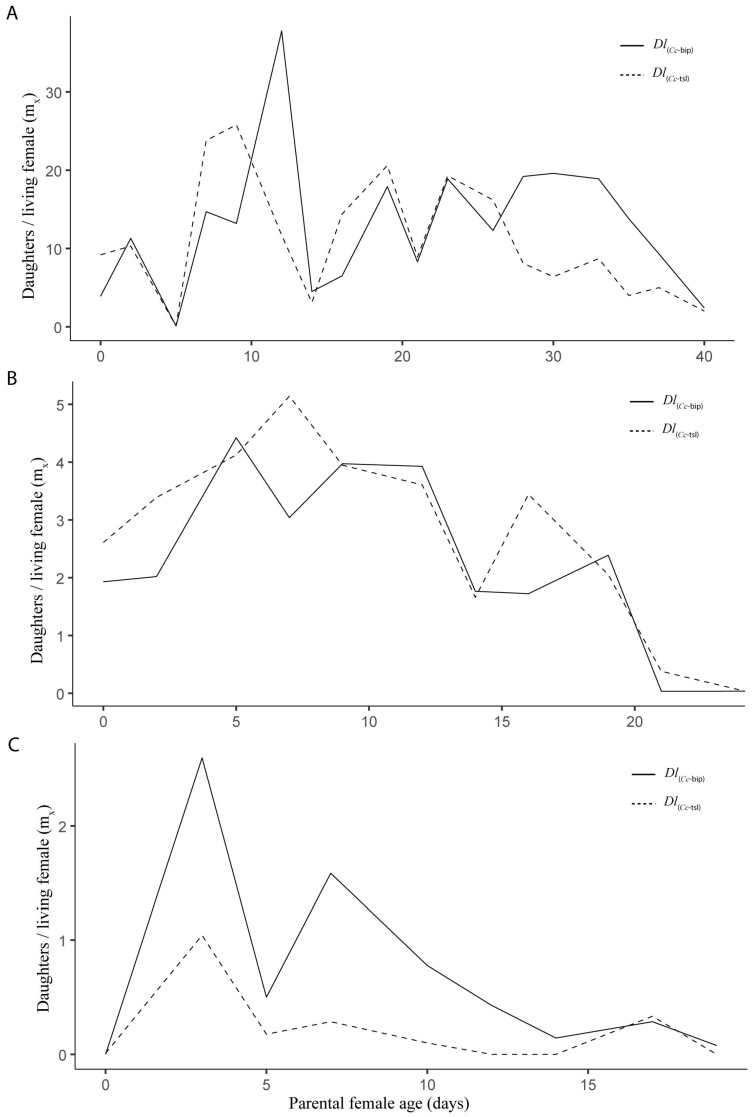
Daily fecundity (*mx*), number of daughters/parental female/day) for two population lines of *D. longicaudata* (*Dl*_(*Cc*-tsl)_ and *Dl*_(*Cc*-bip)_) recorded from semi-field cage trials in spring (**A**), summer (**B**), and autumn (**C**) in the Tulum Valley, San Juan Province, central-western Argentina.

Both *Dl*PLs showed fecundity curves (*mx*) that were significantly similar in spring (*t* = 0.266, *df* = 1, *p* = 0.792) (Figure 6A) and summer (*t* = 0.806, *df* = 1, *p* = 0.436) (Figure 6B). In contrast, a significant difference was found in autumn (*t* = 2.551, *df* = 1, *p* = 0.032) (Figure 6C). Over *Dl*_(*Cc*-tsl)_ females’ lifespan, 0.49 (±0.19) significantly fewer daughters than *Dl*_(*Cc*-bip)_ were produced in autumn. In spring, the *Dl*_(*Cc*-bip)_ females showed five peaks of daughter production between 2 and 28 d old, whereas *Dl*_(*Cc*-tsl)_ females showed four peaks of daughter production between 2 and 24 d old (Figure 6A). In summer, the *Dl*_(*Cc*-bip)_ females showed three peaks of daughter production between 3 and 19 d old, whereas *Dl*_(*Cc*-tsl)_ females showed two pronounced peaks of daughter production between 6 and 16 d old (Figure 6B). In autumn, the *Dl*_(*Cc*-bip)_ females developed two very pronounced peaks of daughter production between 3 and 7 d old, and one slight peak of daughter production at 17 d old, whereas *Dl*_(*Cc*-tsl)_ females developed one pronounced peak of daughter production at 3 d old, followed by a couple of slight increases in the female offspring at 7 and 17 d old (Figure 6C). The parental female age significantly influenced the female offspring yields in both *Dl*PLs during the three tested seasons (spring, *F* = 8.688, e.*df* = 2.942, Ref.*df* = 3.688, *p* = 0.0001, adjusted-*R*^2^ = 0.461; summer, *F* = 4.624, e.*df* = 7.782, Ref.*df* = 8.625, *p* = 0.0084, adjusted-*R*^2^ = 0.626; autumn, *F* = 3.844, e.*df* = 7.216, Ref.*df* = 7.817, *p* = 0.0378, adjusted-*R*^2^ = 0.635). According to the linear model, both T_min_ and RH_max_ were weather factors that fitted to the model when the fecundity of both *Dl*_(*Cc*-bip)_ and *Dl*_(*Cc*-tsl)_ was analyzed (*Dl*_(*Cc*-bip)_, *F* = 16.78 on 2 and 35 *df*, *p* = 7.320 × 10^−7^, adjusted-*R*^2^ = 0.5614; *Dl*_(*Cc*-tsl)_, *F* = 21.95 on 2 and 35 *df*, *p* = 6.636 × 10^−7^, adjusted-*R*^2^ = 0.5311). Both T_min_ and RH_max_ significantly influenced both *Dl*LPs (*Dl*_(*Cc*-bip)_: T_min_, *t* = 2.657, *p* = 0.0119, and RH_max_, *t* = 2.193, *p* = 0.0353; *Dl*_(*Cc*-tsl)_: T_min_, *t* = 6.607, *p* = 1.240 × 10^−7^, and RH_max_, *t* = 3.601, *p* = 0.0009). Female-biased offspring sex ratios were recorded for both *Dl*_(*Cc*-bip)_ and *Dl*_(*Cc*-tsl)_ in spring and summer (1.4:1 and 1.7:1 *Dl*_(*Cc*-bip)_ females/male, respectively; 1.1:1 *Dl*_(*Cc*-tsl)_ females/male in both seasons). A male-biased offspring sex ratio was recorded for both *Dl*LPs in autumn (0.7:1 *Dl*_(*Cc*-bip)_ females/male and 0.5:1 *Dl*_(*Cc*-tsl)_ females/male).

The population increase parameters (*R*_0_, *r*, *T*, and *λ*) of each *Dl*PL varied substantially between different seasons during which the semi-field cage trials were performed (Table 3). Only the *R*_0_ parameter recorded in spring and summer was similar for each *Dl*PL, and between both *Dl*PLs (Table 3). The highest *r* parameter was recorded in summer in both *Dl*PL, and their mean values were substantially similar. The lowest *r* was recorded in autumn for both *Dl*PL, with a substantially higher mean value for *Dl*_(*Cc*-bip)_. (Table 3).

## 4. Discussions

One of the strategic goals of demographic studies on parasitoids is to provide valuable input on their use as potential biological pest control agents and the stability of host–parasitoid systems. The reproductive capacity of females during their lifetime plays a crucial role in understanding the parasitoid population dynamics [28,30]. Such information, as other parasitoid life history traits, is essential to the success of biological control programs, mainly in the context of reducing environmental impact and supporting sustainability [78]. Within this framework, the current study reports a comparative demographic analysis of the exotic parasitoid *D. longicaudata* reared on two different strains of the invasive pest *C. capitata*, one wild-type and one genetically modified. Tests were carried out in the laboratory and, for the first time, under semi-field cage conditions. The latter enables the performance of both *Dl*PLs to be assessed when released at different seasons in the fruit-growing semi-arid region of central-western Argentina. The results underscore important findings as follows: (1) the *Dl*_(*Cc*-tsl)_ was slightly outperformed by the *Dl*_(*Cc*-bip)_ in terms of the female *ex*, *lx* and population growth rates, when both *Dl*PLs were assessed under laboratory conditions; (2) *Dl*_(*Cc*-bip)_ females displayed *lx*- and *ex*-values higher than those recorded for *Dl*_(*Cc*-tsl)_ females in spring, whereas the *mx*-parameter was similar in both *Dl*PLs during spring and summer trials, but it was strongly different between *Dl*PLs in the autumn; (3) both *Dl*LPs recorded high and similar *R*_0_- and *r*-values in spring and summer, but these parameters were low and different between the two *Dl*PLs in autumn; and (4) the T_min_ had a significant influence on the temporal variation in *lx*- and *mx*-parameters in both *Dl*PLs, and the RH_max_ only on the *mx*-parameter in the two *Dl*PLs during semi-field cage studies.

The first finding revealed higher *ex*- and *mx*-values for *Dl*_(*Cc*-bip)_ females than those recorded for *Dl*_(*Cc*-tsl)_ females. In addition, *R_0_*, *r*, and *λ* estimated for *Dl*_(*Cc*-bip)_ were also higher than those for *Dl*_(*Cc*-tsl)_. Among those parameters, the *r* is the most outstanding concerning their importance in assessing parasitoid population dynamics [28,29,31]. Such parameters involve both survival and reproductive data of a parasitoid population, and it is therefore an important indicator for describing the potential population growth over time, under rearing conditions, or when released [23,24,28,79]. More suitable hosts may ensure a high production and better quality of offspring while maintaining acceptable parasitoid rearing costs [80]. Although *Dl*_(*Cc*-bip)_ females displayed a better reproductive capacity under laboratory conditions, it is important to take into account that the results of the current study may be related to the age of the *Dl*PL cohorts used in trials. In this regard, *Dl*_(*Cc*-tsl)_ females were kept under rearing conditions using the *Cc*_tsl_ strain for 50 generations longer than *Dl*_(*Cc*-bip)_. The *D. longicaudata* colony established on the *Cc*_bis_ strain was only 10 generations old when the study was performed. Thus, it appears reasonable to assume that the demographic parameters and population increase values may differ, as shown in this study. Consistent with that thought, some studies on the *D. longicaudata* demography performed in the laboratory point to the effect of time elapsed under rearing conditions using different host species [24,81,82], host stage age [44], or host strains [83]. Older parasitoid cohorts may have lower survival rates, which influences the reproductive rate and overall population growth of that parasitoid species [84,85]. Nevertheless, the potential influence of the *C. capitata* strain cannot be ruled out in the current study, which requires further in-depth research on this topic. Interestingly, the crucial *r*-parameter, which stands for the instantaneous or per capita population growth rate, recorded for both *Dl*PLs, was appreciably higher than that recorded in other laboratory studies on *D. longicaudata* reared on other tephritid host species or on different *C. capitata* strains (see Table 4). The *r*-value recorded for both *Dl*PLs outperformed the highest values of *r* published for *C. capitata*, *A. fraterculus*, and *Bactrocera dorsalis* Hendel (Diptera: Tephritidae) by 2.1- and 2.3-fold, 1.9- and 2.1-fold, and 1.7- and 1.9-fold, respectively (Table 4). Laboratory conditions, larval diets, host larval age, the host species, and/or the medfly strains could probably explain the difference between those values.

The second finding showed interesting data on *lx*-, *ex*- and *mx*-parameters of both *Dl*PLs recorded during field-cage trials. Although in spring the *lx*- and *ex*-parameters recorded for *Dl*_(*Cc*-bip)_ females were higher than those of *Dl*_(*Cc*-tsl)_ females, the *mx* in both *Dl*PLs was statistically similar. Each *Dl*_(*Cc*-bip)_ female and each *Dl*_(*Cc*-tsl)_ female produced 57.6 ± 6.4 and 52.9 ± 3.8 daughters over their lifetime in the spring, respectively. During summer semi-field cage trials, *Dl*_(*Cc*-bip)_ females recorded a slightly higher *ex* than *Dl*_(*Cc*-tsl)_ females, but both *lx*- and *mx*-parameters were similar between the two *Dl*PLs. The production of daughters per living maternal female of both *Dl*_(*Cc*-bip)_ and *Dl*_(*Cc*-tsl)_ during the summer trial was 31.5 ± 1.2 and 34.1 ± 1.8 daughters over their lifetime, respectively. Surprisingly, in the autumn semi-field cage trials, both *ex*- and *mx*-parameters recorded for *Dl*_(*Cc*-bip)_ increased substantially compared to that reported for *Dl*_(*Cc*-tsl)_. However, the *lx* was similar, and females of both *Dl*PLs did not reach 20 d old. Such information is relevant as it highlights two outstanding issues. Firstly, the performance of females from the two *Dl*PLs in terms of their female offspring production was similar under temperate-to-warm natural environmental conditions, with mean daily temperatures between 20 and 22 °C, as recorded during trials in late spring and midsummer. Secondly, *Dl*_(*Cc*-bip)_ females could be more successfully productive than *Dl*_(*Cc*-tsl)_ females under natural conditions with colder temperatures (mean daily temperature around 13 °C), as recorded during early autumn trials. This finding may indicate a higher biological plasticity in *Dl*_(*Cc*-bip)_ females; that is, these females may tolerate a broader temperature range than *Dl*_(*Cc*-tsl)_ females. Data recorded in the autumn showed that the production of daughters per *Dl*_(*Cc*-bip)_ maternal female doubled that of *Dl*_(*Cc*-tsl)_ females up to the first 10 days of the females’ lives. Thus, the mean number of female offspring per living maternal female of both *Dl*_(*Cc*-bip)_ and *Dl*_(*Cc*-tsl)_ during the autumn trial was 4.5 ± 1.0 and 2.1 ± 0.8 daughters over their lifetime, respectively. However, such an assumption must be further tested with new trials under natural conditions throughout autumn for at least two consecutive years. *Diachamimorpha longicaudata* evidently can adapt and establish itself in environments that exhibit significant seasonal variation in temperature, humidity, and precipitation, such as subtropical [44] or semi-desert [50,51] regions, or in Mediterranean climate [42,43], and in tropical environments with a more stable climate [1].

The third finding revealed similar population growth rates in both *Dl*LPs during the spring and summer trials but substantial differences in the autumn trial, when *Dl*_(*Cc*-bip)_ females displayed higher reproductive success than *Dl*_(*Cc*-tsl)_ females. The above is consistent with the *mx*-values recorded in the three seasons and discussed above. The *mx*-parameter is closely linked to the parasitoid population growth, because higher productivity influences the increase in the number of future generations of the parasitoid species [86,87]. Therefore, it was hypothesized that such a population increase improves the parasitoid’s ability to control host populations [88]. In this sense, parameters such as *R*_0_ (females produced per generation) and *r* (maximum potential rate of population growth) are highly influenced by the fecundity [88,89,90]. However, the complexity of the environmental context, including pest features, food availability, crop management practices, and environmental conditions such as temperature, can hide patterns related to the success of biological control in relation to the parasitoid life history traits and consequently affect its population growth rate [89,91]. Despite this, both *R*_0_ and *r*, determined particularly under natural environmental conditions, are key parameters for predicting the potential population increase that females of both *Dl*PLs may develop when used in open-field releases. In this regard, results from the current study revealed that *R*_0_-values in both *Dl*PLs recorded in spring and summer trials were 12- to 15-fold and 49- to 58-fold higher than *R*_0_-values recorded in early autumn for *Dl*_(*Cc*-bip)_ and *Dl*_(*Cc*-tsl)_, respectively. Likewise, the *R*_0_-value less than 1 recorded in autumn for *Dl*_(*Cc*-tsl)_ females indicates a declining population.

In contrast, the *R*_0_-value slightly greater than 1 recorded for *Dl*_(*Cc*-bip)_ females during the autumn trial indicated a population growing on a small scale. The *r*-parameter showed a similar pattern to *R*_0_ about the *Dl*PLs related to the three tested seasons. However, in the summer trial, both *Dl*PLs recorded a substantially higher *r*-value compared to spring and autumn. The *r*-value recorded for *Dl*_(*Cc*-bip)_ in summer was 2.3-fold and 10.6-fold higher than that recorded for spring and autumn, respectively. In the same pattern, the *r*-value recorded for *Dl*_(*Cc*-tsl)_ in summer was 2.3-fold and more than 41-fold higher than those recorded for spring and autumn, respectively. The negative *r*-value recorded for *Dl*_(*Cc*-tsl)_ females in autumn showed a declining population with more individuals dying than being born. Variations in population growth rates within the same *Dl*PL could be related to changes in weather conditions, mainly temperature. Several laboratory studies have demonstrated the significant effect of temperature on the simulated population growth parameters of *D. longicaudata* [39,44,82,92], regardless of the host species used in the test. For instance, the simulated *r*-value for *D. longicaudata* can substantially increase when the temperature increases between 15 and 30 °C [92]. This is closely associated with a highly temperature-dependent *mx*-value in *D. longicaudata* [44], which is described as follows in the fourth finding.

Concerning the four findings, the close relationship between air T_min_ and both *lx* and *mx* recorded for the two *Dl*PLs can be mainly attributed to the decline in the lifespan and reproductive capacity of parasitoid females starting in the early autumn. The coldest environmental conditions in the region under study began during that season. This is consistent with previous authors [39,44,82,92], who have shown through laboratory studies by using life cycle simulation modeling that temperature has a strong effect on the development time, adult survival, and fecundity of *D. longicaudata*. Different nonlinear models predicted 10.0–10.4 °C and 31.0–33.7 °C as lower and upper thermal thresholds for the survival from egg to adult in *D*. *longicaudata* [44,92], and 28.0 °C as the optimum temperature for adult survival [44]. During the semi-field cage trial performed at the study site in the early autumn, the T_min_ ranged between 2.9 and 9.3 °C, i.e., values below the lower thermal threshold determined for *D. longicaudata*. The range of temperatures below 10 °C may clarify the low *lx* rate of *D. longicaudata* females in the first week of the trial, with 75% of parasitoid females dying during this period. On the contrary, during spring and summer trials, the T_min_ ranged from 5.3 °C to 22.0 °C, but with a T_min_ mean close to 14 °C in both seasons. Such a mean value exceeds the lower thermal threshold for *D. longicaudata*. Environmental temperature conditions may explain the high survival rate of *D. longicaudata* females, which surpassed 80% in the first week of semi-field cage trials. Similarly to the above, the *mx* in both *Dl*LPs was influenced by T_min_. Parasitoid females were considerably more fecund in spring and summer, seasons in which the mean T_min_ was 2.6-fold higher than in autumn. Different authors based on laboratory studies point out that the *D. longicaudata* fecundity was higher between 24 °C and 30 °C [39,44,92]. This information matches with a previous study in a fig-producing farm located in San Juan province, where it was found that parasitism on *C. capitata*, related to egg-laying by released *D. longicaudata* females, increased at higher temperatures and relative humidity [50]. Interestingly, a semi-field cage study performed in an area of eastern Spain [42], characterized by a classical Mediterranean climate, pointed out that the parasitism by *D. longicaudata* on *C. capitata* increased with mean temperature but decreased with mean relative humidity. According to such a study [42], the optimal climatic conditions for the *D. longicaudata* activity were 16–24 °C and at 45–60% RH, values that match the mean temperatures and RH recorded in spring and summer during the current study. However, the RH_max_ was also an environmental factor influencing the *mx* of both *Dl*PLs, similar to that reported by Sánchez et al. [50], and the *mx* was mainly conditioned by the T_min_. This is because the RH_max_ had high values, between 69 and 84%, in the three seasons in which trials were carried out. Thus, the mean RH_max_ was higher in early autumn than that recorded in both summer and spring, but the mean T_min_ was lower than the minimum thermal threshold that *D. longicaudata* females can tolerate.

## 5. Conclusions

Results of the comparative laboratory trials may confirm the first hypothesis of this study, whereby *Dl*_(*Cc*-bip)_ females exhibited higher reproductive potential than that of *Dl*_(*Cc*-tsl)_ females. Such findings may optimize the *D. longicaudata* production and quality under mass rearing conditions at the San Juan Biofactory, keeping costs within an acceptable level. Likewise, the results of semi-field cage trials showed high-quality females with high reproductive capacity in both *Dl*PLs. That uncovers a key factor for successful population growth and performance of the parasitoid when used for *C. capitata* biological control. In this regard, the second hypothesis of this research is also supported. Females of both *Dl*PLs showed high population growth rates during spring and summer, seasons in which the highest population peaks of *C. capitata* occur in the study region. That suggests the temperature or relative humidity stress probably canceled out any advantage of *Dl*_(*Cc*-bip)_ females over *Dl*_(*Cc*-tsl)_ females, but during the warmer seasons (spring and summer). This is because this study revealed an engaging, novel, and additional finding. Apparently, *Dl*_(*Cc*-bip)_ females can adapt better to colder environmental conditions than female *Dl*_(*Cc*-tsl)_ females, as they were able to sustain a low population growth rate at least in early autumn. Based on this result, *Dl*_(*Cc*-bip)_ females could be released between early and mid-autumn in fruit-growing areas of San Juan, when *C. capitata* populations are starting to decline. The results reported are important for assessing improvements in *D. longicaudata* mass production at the San Juan Biofactory and for redesigning the parasitoid release schedule throughout Argentina’s irrigated semi-arid fruit-production regions.

## Figures and Tables

**Table 1:** Mean (±SE) air temperature (T_mean_), mean (±SE) maximum air temperature (T_max_), mean (±SE) minimum air temperature (T_min_), mean (±SE) range air temperature (T_range_), mean (±SE) relative humidity (RH_mean_), mean (±SE) maximum relative humidity (RH_max_), mean (±SE) minimum relative humidity (RH_min_), and mean (±SE) range relative humidity (RH_range_) recorded during semi-field cage trials between October and November/2019 (spring), February and March/2020 (summer), and May/2020 (autumn) in the experimental plot of the Plant, Animal, and Food Health Bureau, Rivadavia District, Tulum Valley, San Juan province, central-western Argentina.

	Temperature (°C)				Relative Humidity (%)			
Seasons	T_mean_	T_max_	T_min_	T_range_	RH_mean_	RH_max_	RH_min_	RH_range_
Spring	19.6 ± 0.6	26.2 ± 0.8	13.7 ± 0.6	17.3 ± 0.9	48.3 ± 2.2	69.4 ± 2.8	27.4 ± 2.4	42.4 ± 3.0
Summer	22.0 ± 0.5	30.3 ± 0.7	13.9 ± 0.6	22.7 ± 0.8	51.2 ± 1.0	78.1 ± 1.5	23.6 ± 1.1	54.6 ± 1.5
Autumn	12.7 ± 0.3	20.0 ± 0.6	5.3 ± 0.5	13.6 ± 0.9	62.8 ± 1.1	83.5 ± 2.1	42.0 ± 0.9	41.6 ± 2.4

**Table 2:** Population parameters [Net reproductive rate (*R*_0_), intrinsic rate of increment (*r*), mean generation time (*T*) and finite rate of increase (*λ*)] for two population lines of *D. longicaudata* (*Dl*_(*Cc*-tsl)_ and *Dl*_(*Cc*-bip)_) under constant laboratory conditions [Mean ± standard error (SE), and confidence intervals (CI) with lower and upper bounds (lo–hi)]. Different letters indicate significant differences between values by CI comparison.

	Population Increase Parameters			
Parasitoid Population Lines	*R*_0_ (Females/Female Per Generation)	*r* (Per Day)	*T* (Days)	*λ* (Per Day)
*Dl*_(*Cc*-bip)_	109.80 ± 3.46	0.32 ± 0.00	14.63 ± 0.30	1.37 ± 0.00
	[108.8–122.4] ^a^	[0.29–0.32] ^a^	[14.53–15.82] ^a^	[1.34–1.38] ^a^
*Dl*_(*Cc*-tsl)_	63.94 ± 3.35	0.29 ± 0.00	14.22 ± 0.58	1.33 ± 0.01
	[68.3–81.4] ^b^	[0.24–0.28] ^b^	[15.12–17.50] ^a^	[1.28–1.32] ^b^

**Table 3:** Population parameters [Net reproductive rate (*R*_0_), intrinsic rate of increment (*r*), mean generation time (*T*) and finite rate of increase (*λ*)] for two population lines of *D. longicaudata* (*Dl*_(*Cc*-tsl)_ and *Dl*_(*Cc*-bip)_) under semi-field cage conditions at three different seasons (spring, summer and autumn) [Mean ± standard error (SE), and confidence intervals (CIs) with lower and upper bounds (lo–hi)]. Different letters indicate significant differences between values by CI comparison.

		Population Increase Parameters			
Parasitoid Population Lines	Seasons	*R*_0_ (Females/Female Per Generation)	*r* (Per Day)	*T* (Days)	*λ* (Per Day)
*Dl*_(*Cc*-bip)_	Spring	23.26 ± 3.45 [16.93–30.17] ^a^	0.15 ± 0.02 [0.12–0.20] ^b^	20.67 ± 2.75 [15.74–26.16] ^a^	1.16 ± 0.03 [1.12–1.23] ^b^
	Summer	19.80 ± 3.37 [11.29–25.50] ^a^	0.35 ± 0.09 [0.21–0.56] ^a^	8.42 ± 1.73 [5.45–12.21] ^b^	1.42 ± 0.14 [1.24–1.75] ^a^
	Autumn	1.56 ± 0.72 [0.40–3.09] ^b^	0.08 ± 0.10 [−0.09–0.27] ^c^	5.72 ± 1.94 [3.78–10.82] ^b^	1.08 ± 0.11 [0.92–1.31] ^c^
*Dl*_(*Cc*-tsl)_	Spring	19.76 ± 3.17 [13.61–26.05] ^a^	0.17 ± 0.03 [0.12–0.25] ^b^	17.15 ± 2.52 [12.65–22.15] ^a^	1.19 ± 0.04 [1.13–1.28] ^b^
	Summer	22.48 ± 4.21 [14.41–30.05] ^a^	0.41 ± 0.11 [0.03–0.12] ^a^	7.65 ± 1.69 [4.82–11.77] ^b^	1.50 ± 0.18 [1.27–1.97] ^a^
	Autumn	0.36 ± 0.23 [0.06–0.85] ^c^	−0.23 ± 0.10 [−0.44–−0.04] ^d^	4.50 ± 2.28 [3.19–11.07] ^b^	0.80 ± 0.08 [0.64–0.96] ^d^

**Table 4:** Intrinsic rate of natural increment (*r*) recorded in the literature for *Diachasmimorpha longicaudata* under laboratory conditions at different temperature ranges.

Host Species	Intrinsic Rate of Natural Increment (*r*)	Laboratory-Tested Temperatures (°C)	References
*Anastrepha fraterculus* (Wiedemann)	0.17 ± 0.03	25.0 ± 2.0	[81]
*Ceratitis capitata* (Wiedemann)	0.14 ± 0.02	25.0 ± 2.0	[81]
*C. capitata* (wild strain)	0.098 ± 0.005	22.9 ± 2.9	[82]
*C. capitata* (genetic sexing strain Cast-191)	0.094 ± 0.004	22.9 ± 2.9	[82]
*Bactrocera dorsalis* Hendel	0.003 ± 0.001–0.145 ± 0.001	15–30	[44]
*B. dorsalis*	−0.0240–0.1318	15–30	[39]
*B. dorsalis*	0.12	26.0 ± 2.0	[24]

## Data Availability

The data analyzed in this study are available in the Supplementary Materials (S5–S8), and they can also be found in the CONICET (Argentina) website link: http://ri.conicet.gov.ar/handle/11336/270779. Accessed on 8 September 2025.

## Supplementary Materials

The following supporting information can be downloaded at https://www.mdpi.com/article/10.3390/insects16101031/s1. File S1: Life Table-laboratory; File S2: Life Table-spring; File S3: Life Table-summer; File S4 Life Table-autumn; File S5: Life-table-lab.Rmd; File S6: Life-table-Season.Rmd; File S7: model-lx-beta-analysis.Rmd; File S8: model-mx-analysis.Rmd.
